# A Novel Bunyavirus Discovered in Oriental Shrimp (*Penaeus chinensis*)

**DOI:** 10.3389/fmicb.2021.751112

**Published:** 2021-11-24

**Authors:** Xuan Dong, Tao Hu, Yanbei Ren, Fanzeng Meng, Chen Li, Qingli Zhang, Jiayuan Chen, Jipeng Song, Ruoyu Wang, Mang Shi, Juan Li, Peng Zhao, Cixiu Li, Kathy F. J. Tang, Jeff A. Cowley, Weifeng Shi, Jie Huang

**Affiliations:** ^1^Yellow Sea Fisheries Research Institute, Chinese Academy of Fishery Sciences, Laboratory for Marine Fisheries Science and Food Production Processes, Pilot National Laboratory for Marine Science and Technology (Qingdao), Key Laboratory of Maricultural Organism Disease Control, Ministry of Agriculture and Rural Affair, Qingdao Key Laboratory of Mariculture Epidemiology and Biosecurity, Qingdao, China; ^2^Key Laboratory of Etiology and Epidemiology of Emerging Infectious Diseases in Universities of Shandong, Shandong First Medical University and Shandong Academy of Medical Sciences, Taian, China; ^3^Shandong Provincial Key Laboratory of Animal Biotechnology and Disease Control and Prevention, Shandong Agricultural University, Taian, China; ^4^School of Medicine, Sun Yat-sen University, Guangzhou, China; ^5^Livestock and Aquaculture, CSIRO Agriculture and Food, Queensland Bioscience Precinct, St. Lucia, QLD, Australia; ^6^Network of Aquaculture Centres in Asia-Pacific, Bangkok, Thailand

**Keywords:** bunyavirus, *Penaeus chinensis*, *Wenrivirus*, *Phenuiviridae*, shrimp

## Abstract

Herein, we describe a novel bunyavirus, oriental wenrivirus 1 (OWV1), discovered in moribund oriental shrimp (*Penaeus chinensis*) collected from a farm in China in 2016. Like most bunyaviruses, OWV1 particles were enveloped, spherical- to ovoid-shaped, and 80–115 nm in diameter. However, its genome was found to comprise four segments of (-)ssRNA. These included an L RNA segment (6,317 nt) encoding an RNA-directed RNA polymerase (RdRp) of 2,052 aa, an M RNA segment (2,978 nt) encoding a glycoprotein precursor (GPC) of 922 aa, an S1 RNA segment (1,164 nt) encoding a nucleocapsid (N) protein of 243 aa, and an S2 RNA segment (1,382 nt) encoding a putative non-structural (NSs2) protein of 401 aa. All the four OWV1 RNA segments have complementary terminal decanucleotides (5′-ACACAAAGAC and 3′-UGUGUUUCUG) identical to the genomic RNA segments of uukuviruses and similar to those of phleboviruses and tenuiviruses in the *Phenuiviridae*. Phylogenetic analyses revealed that the RdRp, GPC, and N proteins of OWV1 were closely related to Wēnzhōu shrimp virus 1 (WzSV-1) and Mourilyan virus (MoV) that infect black tiger shrimp (*P. monodon*). Phylogenetic analyses also suggested that OWV1 could be classified into a second, yet to be established, species of the *Wenrivirus* genus in the *Phenuiviridae*. These wenriviruses also clustered with Wenling crustacean virus 7 from shrimps and bunya-like brown spot virus from white-clawed crayfish. Of note there were no homologs of the NSs2 of OWV1 and MoV/WzSV-1 in GenBank, and whether other crustacean phenuiviruses also possess a similar S2 RNA segment warrants further investigation. In addition, we established a TaqMan probe-based reverse-transcription quantitative PCR method for detection of OWV1, and it was detected as 1.17 × 10^2^—1.90 × 10^7^ copies/ng-RNA in gills of 23 out of 32 *P. chinensis* samples without an obvious gross sign. However, the discovery of OWV1 highlights the expanding genomic diversity of bunyaviruses.

## Introduction

The *Bunyavirales* order was established in 2016 to allow the taxonomic assignment of a plethora of new viruses discovered in diverse vertebrates, invertebrates, and plants. The number of members in the order has significantly increased in recent years, especially those discovered by next-generation sequencing, particularly meta-transcriptome (Tang et al., 2018; Abudurexiti et al., 2019; Kuhn et al., 2020). The genomic organization of a typical bunyavirus consists of three segments of (-)ssRNA: large (L), medium (M), and small (S). However, more bunya-like viruses with genomes comprising two up to as many as eight RNAs have been reported (Li et al., 2015; Shi et al., 2016, 2018; Tang et al., 2018). For example, the thrips-transmitted tenuiviruses of plants contain four to six RNAs (Abudurexiti et al., 2019; Kuhn et al., 2020), highlighting the continually increasing genetic diversity of bunyaviruses. However, the biological significance and function of proteins encoded by some of these RNA segments remain unknown.

Historically, arthropod-vectored bunyaviruses were discovered due to the diseases that they caused in vertebrates and plants. Similarly, bunya-like viruses from crabs and shrimps cause diseases associated with the inhibition of hemolymph clotting and elevated mortality during culture (Bang, 1971; Corbel et al., 2003; Cowley et al., 2005a,b; Sellars et al., 2006; Oanh et al., 2011; Cowley, 2020). However, information on the genome makeup and sequences of crustacean bunyaviruses only started to emerge in recent years (Cowley et al., 2005b; Li et al., 2015; Cowley, 2016; Sakuna et al., 2018; Bojko et al., 2019; Grandjean et al., 2019).

The first shrimp bunyavirus discovered was Mourilyan virus (MoV) (Cowley et al., 2005b; Cowley, 2016). It was frequently detected with RT-PCR in black tiger shrimp (*Penaeus monodon*) and kuruma shrimp (*P. japonicus*), but less frequently detected in banana shrimp (*P. merguiensis*) inhabiting eastern Australia (Cowley et al., 2005a,b). Histology and *in situ* hybridization showed that it had a tropism encompassing all tissues of mesodermal and ectodermal origins, including the lymphoid organ, gills, connective tissues, heart, and the nerve cord and ganglia (Cowley et al., 2005a). In *P. japonicus*, elevated MoV infection loads were associated with gradually increased mortality in shrimps reared in tanks following their grow-out in farm ponds (Sellars et al., 2006). Its pathogenic potential has been confirmed recently in experimental challenge trials (Cowley, 2020).

In particular, two bunyaviruses, Wēnzhōu shrimp virus 1 (WzSV-1) with genome composed of three RNA segments and Wēnzhōu shrimp virus 2 (WzSV-2) with two RNAs, were identified from tissue samples of *P. monodon* and ridgetail prawn (*Palaemon carinicauda*), respectively, collected from Wenzhou of Zhejiang Province, China (Li et al., 2015). Furthermore, a recent publication has shown that MoV might be a distinct variant of WzSV-1 due to the high genome similarity, and the genomes of both viruses comprise four RNA segments, with the second small segment (designated S2) encoding a presumed non-structural protein (NSs2) (Cowley, 2021). As for WzSV-2, phylogenetic analyses of the RNA-directed RNA polymerase (RdRp) and glycoprotein sequences showed that it was closely related to athtab bunya-like virus in freshwater redclaw crayfish (*Cherax quadricarinatus*) collected from Atherton Tableland in northern Australia (Sakuna et al., 2018) and some other crustacean bunyaviruses (Shi et al., 2016).

Herein, we describe the genome sequence, morphological features, and phylogenetic relationships of a novel bunyavirus discovered in *P. chinensis* sampled from a farm in China in 2016. Based on the evidence obtained in the present study, we suggest a name oriental wenrivirus 1 (OWV1), and also propose a binomial name *Wenrivirus orientalis* for the species to classify OWV1 (Siddell et al., 2019). Similar to MoV and WzSV-1, OWV1 also possessed an additional small RNA segment, S2, which encoded a putative non-structural (NSs2) protein sharing limited amino acid similarity to MoV and WzSV-1.

## Materials and Methods

### Shrimp Samples and Microscopic and Molecular Diagnosis

In 2016, we collected 200 *P. chinensis* individuals (12–14 cm in body length) from a hatchery in Shandong Province, China, and transported them alive (about two and a half hours) to the Yellow Sea Fisheries Research Institute, Chinese Academy of Fishery Sciences for use in bioassays. Within 2 weeks of being stocked across several aquaria, the shrimp displayed evidence of morbidity without typical clinical signs but suffered from ∼20% mortality from unknown causes. Cephalothorax tissues were sampled from the moribund shrimp to screen pathogens using transmission electron microscopy (TEM) and various PCR/RT-PCR methods. Total RNA and DNA were separately extracted from three pooled samples, and each pooled sample included five cephalothorax tissues. The molecular diagnosis of shrimp pathogens, including white spot syndrome virus (WSSV), infectious hypodermal and hematopoietic necrosis virus (IHHNV), Taura syndrome virus (TSV), infectious myonecrosis virus (IMNV), *Enterocytozoon hepatopenaei* (EHP), and acute hepatopancreatic necrosis disease (AHPND)-causing *Vibrio parahaemolyticus* (*Vp*_AHPND_), was performed using RT-PCR or PCR as previously described (Dangtip et al., 2015; OIE, 2015; Jaroenlak et al., 2016; Chen et al., 2018).

### Transmission Electron Microscopy

Small pieces (∼1 mm^3^) of the lymphoid organ, hepatopancreas, and gills were sampled and fixed in the TEM fixative (2% paraformaldehyde, 2.5% glutaraldehyde, 160 mM NaCl, and 4 mM CaCl_2_ in 200 mM PBS, pH 7.2) to screen any pathogens. Ultrathin sections were cut, mounted on collodion-coated grids, stained with aqueous uranyl acetate/lead citrate using standard procedures, and examined using TEM at the Equipment Center of the Medical College, Qingdao University.

### Virus Purification

Approximately 3 g soft cephalothorax tissues excluding hepatopancreas were sampled from the moribund shrimp and homogenized in 10 mL TNEP (100 mM Tris-HCl, 400 mM NaCl, 10 mM EDTA, pH 7.4) containing 200 μL protease inhibitor (0.675 g PMSF in 40 mL isopropyl alcohol) using a TissueLyser (Qiagen). The homogenate was clarified by low-speed centrifugation, and the supernatant was then centrifuged at 10,000 × *g* for 30 min at 4°C. The pellet was homogenized again in 10 mL TNEP and centrifuged at 8,000 × *g* for 20 min at 4°C. The supernatants were then combined and filtered sequentially through 0.45 μm and 0.22 μm membrane syringe filters to remove bacteria before being ultra-centrifuged in a P90AT rotor at 130,000 × *g* for 170 min at 4°C (CP100WX Ultracentrifuge, Hitachi, Japan). The pellets were resuspended in the TN buffer (100 mM Tris-HCl, 400 mM NaCl, pH 7.4) and loaded onto a sucrose density gradient prepared by layering equal volumes of 20.0, 31.5, 43.0, 54.5, and 66.0% (w/v) sucrose and ultra-centrifuged in a P40ST rotor at 130,000 × *g* for 170 min at 4°C. The density gradients were fractionated. Aliquots of the band were diluted with TN buffer and ultra-centrifuged again in a P90AT rotor at 130,000 × *g* for 120 min at 4°C. The pellets obtained were then resuspended in a total volume of 1 mL TN buffer. Drops of the resuspended materials were placed unto grids, negatively stained with 2% phosphotungstic acid (PTA) (pH 6.5), and examined using TEM (HT7700 electron microscopy, Hitachi, Japan) operating at 80 kV.

### RNA Extraction and Transcriptome Sequencing

Total RNA was extracted from the viral extracts using TRIzol Reagent (Invitrogen), and ribosomal RNA (rRNA) was depleted using a Ribo-zero™ rRNA Removal Kit (Epicenter, United States). RNA sequencing libraries were generated from the rRNA-depleted RNA using NEBNext^®^ Ultra™ Directional RNA Library Prep Kit for Illumina^®^ (NEB, United States). Nest generation sequencing (NGS) was performed on the Illumina Hiseq platform, employing a 150 bp paired-end sequencing strategy. This step and the subsequent bioinformatics analyses were performed as described previously (Dong et al., 2020).

### Viral Genome Verification and Sequence Analysis

To obtain the full-length genome sequence, we designed PCR primers based on the consensus contigs assembled from the Illumina Hiseq reads (Table 1). RNA was extracted from purified virions using a UNlQ-10 Column Trizol Total RNA Isolation Kit (Sangon Biotech, Shanghai). cDNA was synthesized with random primers using the M-MuLV First Strand cDNA Synthesis Kit (Sangon Biotech, China) and was then amplified by PCR using LA Taq (Takara Bio, Japan). To determine the 5′ and 3′ terminal sequences of RNA segments, we conducted rapid amplification of cDNA ends (RACE) using a 5′/3′ RACE kit (Invitrogen). The open reading frames (ORFs) in the RNA segments were predicted using Geneious (version 11.1.5) and annotated using the Conserved Domain Database (CDD) (Marchler-Bauer et al., 2007).

**TABLE 1:** Primer sequences used for 5′-RACE, 3′-RACE, and PCR.

Primers	5′–3′
L-1F	CCGGGTGTGTTCTAATGAATCTAC
L-1R	CATGGATGAAGGAAATGGGTG
L-2F	ATATTGGGACGCCCCCTC
L-2R	CGACTCTGATGGAGACTACCTGTT
L-3F	CTGTTTCCTCCGGGGTATCTC
L-3R	CGATCAGTATTGTAGCAGTGCCTT
L-4F	CCTAGTGTGTTATTCACACAAGCATT
L-4R	GTTCAAGCTGCCTGAGGAACAT
L-5F	CTATGAAACCATGTATGTAACTAG
L-5R	TTTGAGGTACATGAAATACGTGTGTT
L-6F	ACATAAGTACCCTACATAGTTTAGGTTCAC
L-6R	TCACAGAGTGGACATAGCCTTAGAA
L-7F	GACATTCAGCTTATTACTATCGAGGA
L-7R	CTTATGACTATGAGCTGAGGGAGAG
L-8F	TCATCCTCCTCATGGAAAGATACTC
L-8R	GGTGTTGTAGTGTTGCATTGGAA
M-1F	GTAATCAATGATCGTTTAGTTGGTCTT
M-1R	ATCTTTACAGTGGGATTGGAGGTT
M-2F	TCCAAGTCCTTAACATAGAAGCAGTC
M-2R	TATGGATTAGGGGCAACATTGAC
M-3F	CCAGGAGAAACAGTGTGGATTG
M-3R	GCTTGCTGCCAACAATGCT
M-4F	TTGGGGTGATGGAATAGGTTG
M-4R	CGGGAGCTACAAGTCTGCCAT
S1-1F	ACAGTGGTAAGAAGGCAGACAAC
S1-1R	GAGAGCCCAGGGTGATAAACA
S1-2F	TGAAGGAAGTGGCAGCAGAGT
S1-2R	TAACAATCAGAATGCTTATGGTTTG
S2-1F	GTATAGTCAGGCAGTATGCGATTG
S2-1R	CATCTGCCAAGATGCTCTACCA
S2-2F	ACTATATGAGCTGCAGTCTGATCGA
S2-2R	TGGGATGAAGGAATGATGACTGT
S2-3F	TGATCCCAGTACAGGTTCGATG
S2-3R	GATGCCATGCCAGGAATACA
5′adaptor	GCTGTCAACGATACGCTACGTAACG GCATGACAGTGGGIIGGGIIGGGIIG
3′adaptor	GCTGTCAACGATACGCTACGTAACG GCATGACAGTGTTTTTTTTTTTTTTTTTT
5.3′outer	GCTGTCAACGATACGCTACGTAAC
5.3′inner	GCTACGTAACGGCATGACAGTG
S1-3′RACE-F1	CCTGTGTGTGACTTTGAAATGATGGAGC
S1-3′RACE-F2	TCGCTCTCCTCCTGCTTGTGCTCA
S2-3′RACE-F1	TTCTTAACAACCCCCCTGATGGGATACT
S2-3′RACE-F2	TGTGGTTCTGCTCATTGCCCAGGTA
S1-5′RACE-R2	TTCTTCATCAAGGCTGCAACAATGACCT
S1-5′RACE-R1	GCAGATGTCCTCCTTGAGCGTGTTTG
S1-5′RACE-RT2	GCATAGGTCCCCTCGTCT
S1-5′RACE-RT1	GACAGTTGATCATCTCCGTAT
S2-5′RACE-R2	ACATAAAGCTGGAATCAGGGGGAGGAA
S2-5′RACE-R1	AACTCCTGCATTCTGTTTAATGGTTGGCT
S2-5′RACE-RT2	CTATGGAGCTAGATGACTCTCACAAG
S2-5′RACE-RT1	TCTTTCCTCCTATGGCAACTCAG

### Phylogenetic Analyses

BLASTx of the deduced RdRp, glycoprotein precursor (GPC), and nucleocapsid (N) protein sequences against the GenBank database was performed, and the top BLASTx hits to OWV1 were downloaded. Multiple sequence alignment was performed using MAFFT (Nakamura et al., 2018), and the conserved domains were extracted using TrimAl (Capella-Gutierrez et al., 2009) with a heuristic selection of the automatic method based on similarity statistics (-automated1). Phylogenetic analyses were performed using PhyML3.1 (Guindon et al., 2010), with 1,000 bootstrap replicates and the LG amino acid substitution model.

### TaqMan Probe-Based Real-Time RT-qPCR for the Detection of Oriental Wenrivirus 1

We developed a reverse-transcription quantitative real-time polymerase chain reaction (RT-qPCR) targeting the S1 fragment. The product fragment (71 bp) was amplified using the forward primer OWV1-S1-F (5′-CCG ACA TGG ATG CGT TCA-3′), the reverse primer OWV1-S1-R (5′-CAA GGC TGC AAC AAT GAC CTT-3′), and the TaqMan probe (5′-6FAM-CGC AGA CAT CCA GTT CCA GGG CTT T-TAMRA-3′). The TaqMan probe-based RT-qPCR for OWV1 (TaqMan-RTqPCR-OWV1) in a 20 μL mixture, containing 10 μL Probe 1-step RT-qPCR 5G Premix (with 0.08 mM dNTP, 0.01 mM Mg^2+^, mutated MMLV reverse transcriptase, TOROIVD 5G DNA polymerase, RNase inhibitor, reaction buffer, and stabilizer, etc.) (TOROIVD^®^, China), 0.5 μM OWV1-S1-F and OWV1-S1-R primers each, 0.2 μM probe, and 1 μL RNA template. The amplification reaction was performed using a CFX96 Quantitative Fluorescence Instrument (BIO-Rad, California, United States), and the reaction procedure was initiated at 52°C for 5 min and 95°C for 10 s, followed by 40 cycles of 95°C for 10 s and 60°C for 20 s.

## Results

### Screening the Moribund *P. chinensis* for Known Shrimp Pathogens

DNA and RNA extracted from soft tissues of cephalothoraxes of the moribund *P. chinensis* individuals were pooled into three samples, with each including five cephalothorax tissues. Commonly known shrimp pathogens were screened using various PCR or RT-PCR tests, including white spot syndrome virus (WSSV), infectious hypodermal and hematopoietic necrosis virus (IHHNV), Taura syndrome virus (TSV), infectious myonecrosis virus (IMNV), *Enterocytozoon hepatopenaei* (EHP), and acute hepatopancreatic necrosis disease (AHPND)-causing *Vibrio parahaemolyticus* (*Vp*_*AHPND*_). Among the three pooled samples, one sample tested positive for WSSV.

### Transmission Electron Microscopy Examination

**FIGURE 1:**
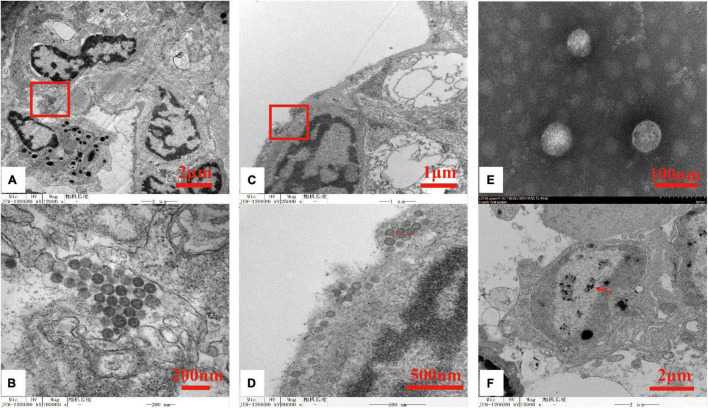
Transmission electron micrographs of oriental wenrivirus 1 (OWV1) and white spot syndrome virus (WSSV) virions. **(A)** Section of the lymphoid organ, bar = 2 μm; **(B)** high magnification of the lymphoid organ section shows the accumulation of OWV1 particles, bar = 200 nm; **(C)** section of gill, bar = 1 μm; **(D)** high magnification of the gill section shows the presence of OWV1 virions, bar = 500 nm; **(E)** purified OWV1 virions stained with 2% phosphotungstic acid (PTA), bar = 200 nm; **(F)** TEM of the ultrathin section revealed the presence of rod-shaped WSSV virions (red arrow), bar = 100 nm.

TEM examination of the ultrathin lymphoid organ and gill tissue sections from moribund *P. chinensis* identified spherical- to ovoid-shaped virions (80 nm∼115 nm in diameter) in the cytoplasm of the lymphoid cells (Figures 1A,B) and branchial cells (Figures 1C,D). In addition, electron microscopy of the negatively stained OWV1 particles purified from soft cephalothorax tissues identified virions morphologically similar to those detected in the infected cells (Figure 1E). Consistent with the PCR detection data, TEM also revealed the presence of WSSV particles (Figure 1F).

### Genome Characterizations of Oriental Wenrivirus 1

A total of 81,931,726 raw reads were obtained from the NGS data of the viral extracts, including 76,860,336 clean reads, which were *de novo* assembled using Trinity. BLASTx searches of the ORFs predicted in the assembled contigs identified three contigs showing homologies to the L, M, and S proteins of bunyaviruses.

In detail, 71,968 reads were mapped back to the L RNA segment (coverage: 1,482 ± 639), 48,574 mapped to the M RNA segment (coverage: 2,139 ± 1,051), and 8,038 mapped to the S RNA segment (coverage: 924 ± 477). To confirm the 3′ and 5′ terminal sequences of each RNA segment, we designed primers for Sanger sequencing to generate 5′- and 3′-RACE clones (Table 1). Sanger sequencing of the clones verified the contigs obtained from NGS. The conserved reverse complementary decamer sequences on the two ends were: 5′-ACACAAAGAC…and …GUCUUUGUGU-3′. These inverted terminal repeat sequences were identical to those of MoV and WzSV-1 (Li et al., 2015; Cowley, 2021) and similar to those of the closely related terrestrial uukuviruses and phleboviruses (Abudurexiti et al., 2019; Kuhn et al., 2020). Like other bunyaviruses, the inverted terminal repeats of OWV1 likely form short panhandles found in bunyaviruses that are important for the N protein binding and nucleocapsid assembly (Mir and Panganiban, 2005).

**FIGURE 2:**
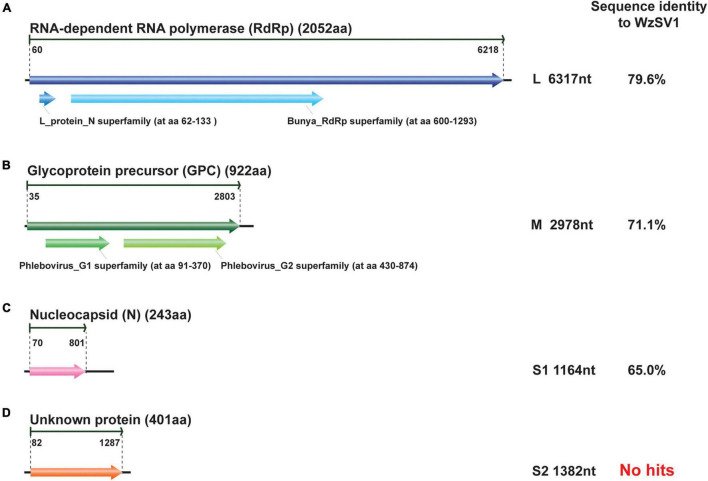
Schematic presentation of the genomic RNA segments and conserved domains of oriental wenrivirus 1 (OWV1). The amino acid sequence identity of encoding protein was compared to that of Wēnzhōu shrimp virus 1 (WzSV-1). The genomic structure was predicted using Geneious, and the conserved protein domains were predicted using CDD.

BLASTp analyses revealed that the OWV1 RdRp (2,052 aa) encoded by the L RNA segment (6,317 nt) (Figure 2A) was most similar to the RdRp of WzSV-1/MoV (79.6%/79.4% identity; *E*-value = 0; query coverage: 100%/100%), followed by Wenling crustacean virus 7 (WCV7, 34.3% identity; *E*-value = 0, query coverage: 95%) and bunya-like brown spot virus (BBSV; 36.7% identity; *E*-value = 0, query coverage: 79%) (Li et al., 2015; Grandjean et al., 2019). Moreover, CDD search found an endonuclease domain at the N-terminus of the predicted RdRp of OWV1 (L_protein_N superfamily domain cl20015; aa 62-133; *E*-value ≤ 1.71e-11) and also a Bunya_RdRp superfamily domain cl20265 (aa 600-1293; *E*-value ≤ 9.41e-97).

The GPC glycoprotein (912 aa), encoded by the M RNA segment (2,978 nt) of OWV1, possessed two transmembrane domains: the Phlebovirus_G1 superfamily domain cl06326 (aa 91-370; *E*-value ≤ 4.34e-13) and the Phlebovirus_G2 superfamily domain cl09431 (aa 430-874; *E*-value ≤ 1.35e-35) (Figure 2B). These domains were one of the specific characterizations of the genus *Phlebovirus*, responsible for anchoring these glycoproteins to the viral envelope (Halldorsson et al., 2016). BLASTp searches revealed that the OWV1 GPC protein was most similar to those of WzSV-1/MoV (71.1%/70.8% identity, *E*-value: 0, coverage: 99%/99%).

Similarly, the nucleoprotein (N) protein (243 aa) encoded by the S RNA segment (1,164 nt) was most similar to those of WzSV-1/MoV (65.0%/65.2% identity; *E*-value ≤ 2e-99, coverage: 90%/92%), followed by BBSV (32.0% identity; *E*-value ≤ 3e-19, coverage: 80%) (Figure 2).

However, it was reported that MoV contained an additional small (S2) RNA segment (1,364 nt) encoding a putative non-structural protein (NSs2; 394 aa) (Oanh et al., 2011; Cowley, 2016). BLASTx searches were repeated using contigs assembled from the OWV1 library, which successfully identified a near-complete RNA segment encoding a homolog of the MoV/WzSV-1 NSs2 protein. A total of 24,110 reads (coverage: 2,508 ± 1,572) were mapped to this contig. In addition, 5′ and 3′ RACE showed that the fourth RNA segment also possessed the complementary decamer terminal sequence identical to those present in the other three RNA segments. Unlike structural proteins, however, the NSs2 protein (401 aa) encoded by the fourth RNA segment (S2; 1,382 nt) of OWV1 was moderately similar to those of WzSV-1/MoV (57.1%/56.6% identity). Apart from the NSs2 protein of MoV/WzSV-1, neither BLASTp nor CDD searches found more homologs of the OWV1 NSs2 protein, whose biological functions thus remain unknown.

### Phylogenetic Analysis

**FIGURE 3:**
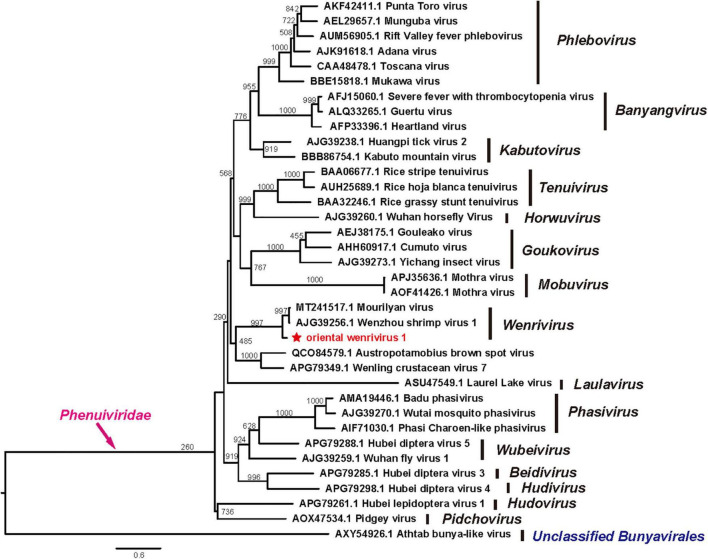
Phylogenetic tree of the RdRp protein sequences of oriental wenrivirus 1 (OWV1) and representative viruses in the family *Phenuiviridae*. OWV1 is highlighted with a red star. Scale bar represents the number of amino acid substitutions per site.

Phylogenetic analyses of the RdRp sequences of OWV1 and other phenuiviruses revealed that OWV1 fell within the genus *Wenrivirus* together with WzSV-1/MoV (Figure 3). However, other related viruses, e.g., WCV7, BBSV, and two phenuiviruses discovered in different crustacean hosts (Li et al., 2015; Grandjean et al., 2019), did not cluster together with OWV1. Similarly, phylogenetic analyses of the GPC and N protein sequences also revealed that OWV1, WzSV-1, and MoV clustered and formed a well-supported lineage (Supplementary Figures 1, 2).

### Detection Oriental Wenrivirus 1 With TaqMan Probe-Based Real-Time Polymerase Chain Reaction

The TaqMan-RTqPCR-OWV1 method was established using OWV1 standard templates in nine concentrations from 1 × 10^1^ to 1 × 10^9^ copies/μL. The method calculated the logarithmic OWV1 copies per ng total RNA (copies/ng-RNA) in inverse proportion to the threshold cycles (CT) with a slope of -4.118 and an intercept of 48.66. The established standard curve had a correlation coefficient of 0.994. Three parallel CT values of each sample are nearly the same, indicating that the intra-assay repeatability was reliable. We collected 32 *P. chinensis* samples from two markets, which had no obvious gross sign when sampling. After being tested with TaqMan-RTqPCR-OWV1, 23 samples were positive for OWV1 at 1.17 × 10^2^—1.90 × 10^7^ copies/ng-RNA of OWV1 in gills of *P. chinensis*.

## Discussion

We report a novel bunyavirus discovered in moribund farmed oriental shrimp *P. chinensis* in China. The morphology and genome sequence of the novel virus revealed that it was mostly related to the wenrivirus MoV and its geographic variant WzSV-1. The difference in its genome sequence from the known virus species and the phylogenetic analyses of the RdRp sequences of OWV1 and other phenuiviruses support it to be a member of new virus species under the genus *Wenrivirus*. We then suggest naming the new virus as OWV1 from the common host name (i.e., oriental shrimp). Consecutively, we intend to prepare and submit an official taxonomic proposal to the ICTV to classify OWV1 as a member of a new species named *Wenrivirus orientalis* to fit recently adopted binominal species nomenclature (Siddell et al., 2019).

OWV1 possesses several distinct molecular features. Firstly, the number of RNA segments of the OWV1 genome is four rather than three, which is the same as that of MoV/WzSV-1 but different from most described bunyaviruses. The existence of a fourth RNA segment in OWV1 expanded the genome flexibility and diversity among bunyaviruses and might also represent the genome characteristics of the genus *Wenrivirus*. Secondly, all the four OWV1 RNA segments possessed complementary terminal decamer sequences (5′-ACACAAAGAC and 3′-UGUGUUUCUG) identical to the genomic RNA segments of uukuviruses and similar to those of phleboviruses and tenuiviruses in the *Phenuiviridae*. The conservation of 5′, - 3′ termini may be the characteristic of viruses whose genomes have multiple RNA segments. Thirdly, OWV1 lacks a variable GA-rich repeat sequence found in the S1 RNA segment of MoV/WzSV-1 (Cowley, 2021). Moreover, as either BLASTp or CDD searches failed to identify homologous proteins to the OWV1 NSs2, the potential function of this protein remains unclear, which warrants further research.

The hosts of the viruses of the family *Phenuiviridae* include insects, mammals, crustaceans, and plants. Most members in genera *Phlebovirus*, *Bandavirus*, and *Kabutovirus* are insect-borne mammalian viruses, and viruses in the genus *Tenuivirus* are insect-borne plant viruses (Supplementary Table 1). The ecological relationship between vampire insects and mammals provides an opportunity for the adaptive evolution of arboviruses. However, no mammalian host has been reported for all known viruses in the family *Phenuiviridae* identified from crustaceans. Notably, Otter fecal bunyavirus identified from feces of a wild Eurasian otter (*Lutra lutra*) that feeds on aquatic animals had a close relationship with Wenling crustacean virus 7 (Bodewes et al., 2014). Furthermore, some crustaceans, such as krill, copepods, and amphipods, are the primary food sources for some marine mammals (SPE, 2021). In addition, some other crustaceans are parasites of mammals, such as whale lice (genus *Cyamus*) and whale barnacles (genus *Cryptolepas*) (Murase et al., 2014). Therefore, crustaceans may be a key linkage of virus transmission among marine mammals establishing virus transmission circles through two possible pathways: i) mammal feeding on crustaceans—crustaceans feeding on mammal feces pathway, or ii) mammal—crustacean parasites biting—mammal pathway in the marine environment. Along with more and more bunyaviruses identified from crustaceans, the arboviral transmission chain-like relationship between crustaceans and marine mammals may be noteworthy.

Crustaceans are one of the most important economic aquatic animals. The world exports of shrimp and prawn products exceeded 23 billion dollars, accounting for 16% of the global fishery commodity trades in 2018 (FAO, 2020). While OWV1 was detected in a *P. chinensis* cohort experiencing morbidity and mortality, it should be noted that co-infection of WSSV and OWV1 was found in this case. In particular, OWV1 of 1.17 × 10^2^ - 1.90 × 10^7^ copies/ng-RNA was detected in gills of *P. chinensis* without an obvious gross sign. Rajendran et al. (2006) reported the detection of up to > 10^4^ copies/ng-RNA (> 10^7^ copies/μg-RNA in the paper) of MoV in lymphoid organs of challenged *P. japonicus* associated with an increase of lymphoid spheroids. It has been experimentally confirmed that MoV infection can cause gradually accumulating mortality in *P. japonicus* (Cowley, 2020). However, Oanh et al. (2011) confirmed that *P. monodon* can tolerate a high-level MoV infection up to > 10^6^ copies/ng-RNA (>10^9^ copies/μg-RNA in the paper) in gills and that mortalities of the mid-crop mortality syndrome (MCMS) outbreaks are associated with the co-infection with gill-associated virus (GAV) rather than MoV. Therefore, whether OWV1 is responsible for or contributes to the pathology and the reduction in shrimp production clearly warrants further study.

## Data Availability Statement

The full-length genome of WvO has been deposited in GenBank under accession numbers: MK335503 (L, 6,317 nt), MK335504 (M, 2978 nt), MK335505 (S, 1,164 nt), and MT040832 (S2, 1,382 nt).

## Author Contributions

XD, WS, and JH designed the study and wrote the manuscript. YR, FM, CL, JCh, JS, and RW performed the biological experiments. XD, TH, JL, MS, and WS assembled preliminary sequences and analysis. QZ, PZ, CL, JCo, and KT discussed the results and modified the manuscript. All authors reviewed the manuscript.

## Conflict of Interest

The authors declare that the research was conducted in the absence of any commercial or financial relationships that could be construed as a potential conflict of interest.

## Publisher’s Note

All claims expressed in this article are solely those of the authors and do not necessarily represent those of their affiliated organizations, or those of the publisher, the editors and the reviewers. Any product that may be evaluated in this article, or claim that may be made by its manufacturer, is not guaranteed or endorsed by the publisher.

## Supplementary Material

Supplementary Figure 1 Phylogenetic analysis of glycoprotein sequences of oriental wenrivirus 1 (OWV1) and representative viruses in the family *Phenuiviridae*. OWV1 is highlighted in red and with a red star.

Supplementary Figure 2 Phylogenetic analysis of nucleoprotein sequences of oriental wenrivirus 1 (OWV1) and representative viruses in the family *Phenuiviridae*. OWV1 is highlighted in red and with a red star.

Supplementary Table 1 Virus-host relationship of *Phenuiviridae*.
