# Supplementary figures and images for: A Novel Bunyavirus Discovered in Oriental Shrimp (Penaeus chinensis)
Source: Front Microbiol. 2021 Nov 24;12:751112. doi: 10.3389/fmicb.2021.751112 (PMC8652140; doi:10.3389/fmicb.2021.751112)

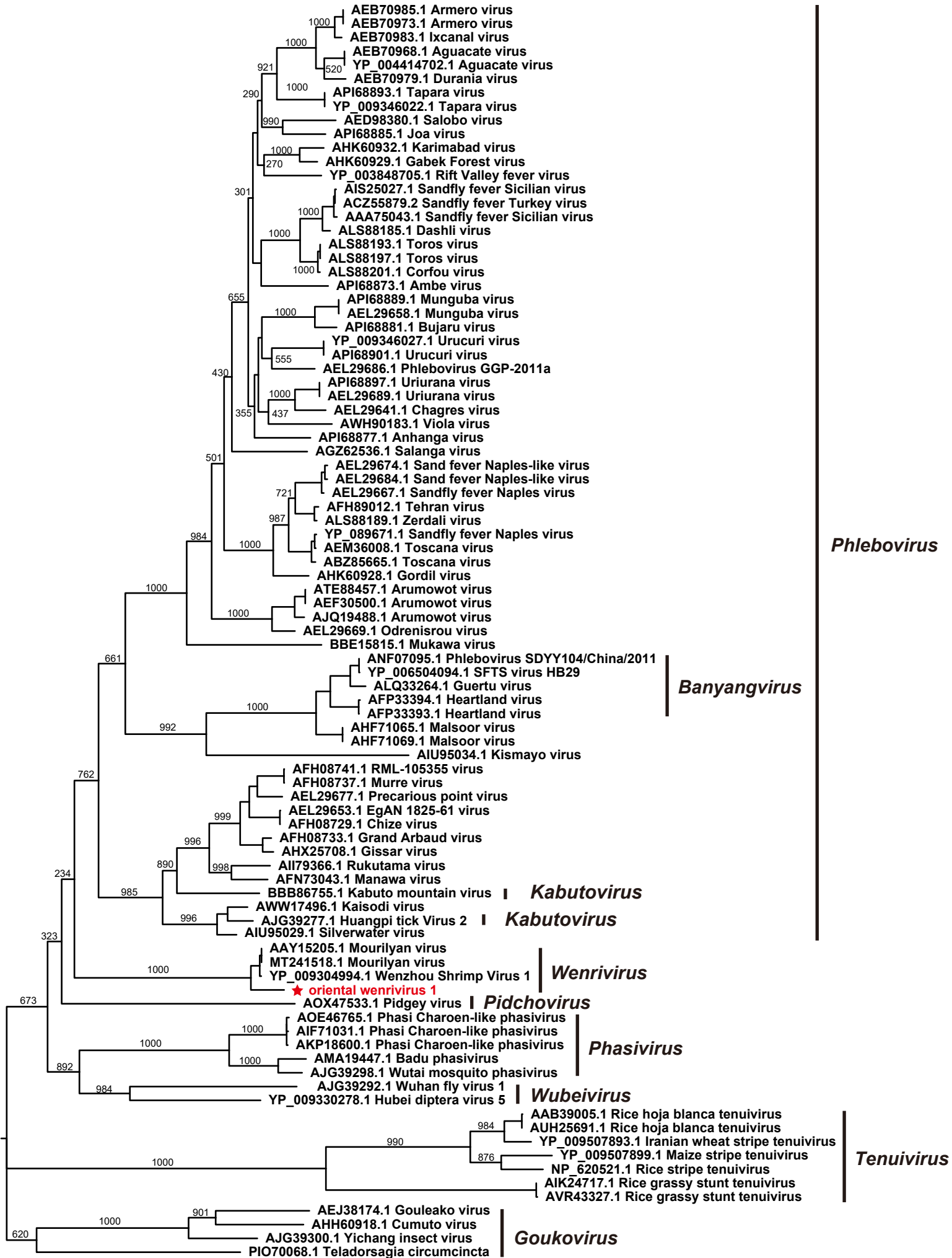

Supplement: Supplementary Figure 1 — Phylogenetic analysis of glycoprotein sequences of oriental wenrivirus 1 (OWV1) and representative viruses in the family Phenuiviridae. OWV1 is highlighted in red and with a red star. [file Image_1.PDF]

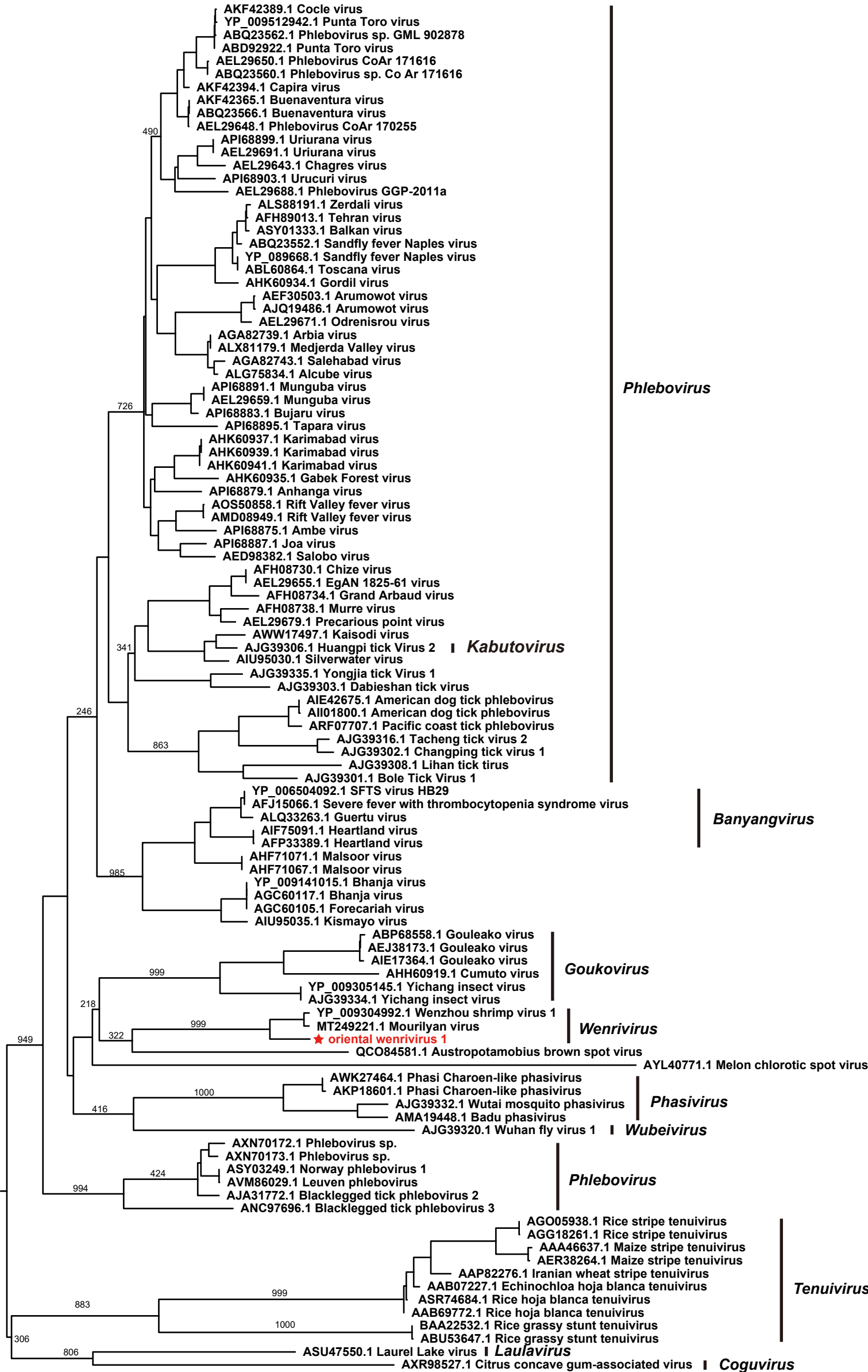

Supplement: Supplementary Figure 2 — Phylogenetic analysis of nucleoprotein sequences of oriental wenrivirus 1 (OWV1) and representative viruses in the family Phenuiviridae. OWV1 is highlighted in red and with a red star. [file Image_2.pdf]
